# Device activism and material participation in healthcare: retracing forms of engagement in the #WeAreNotWaiting movement for open-source closed-loop systems in type 1 diabetes self-care

**DOI:** 10.1057/s41292-022-00278-4

**Published:** 2022-04-22

**Authors:** Bianca Jansky, Henriette Langstrup

**Affiliations:** 1 Ethics of Medicine, Medical Faculty, University of Augsburg, Stenglinstraße 2, 86156 Augsburg, Germany; 2 Institute for Sociology, Ludwig-Maximilians-University, Munich, Germany; 3 Center for Medical Science and Technology Studies, Copenhagen University, Copenhagen, Denmark

**Keywords:** Patient activism, Material participation, Patient innovation, Open-source, Patient collectives, Type 1 diabetes

## Abstract

The #WeAreNotWaiting movement is a global digital health phenomenon in which people with diabetes, mainly type 1 diabetes (T1D), engage in the development and usage of open-source closed-loop technology for the improvement of their “chronic living” (Wahlberg et al. 2021). The characteristics of a digitally enabled and technologically engaged global activist patient collective feed into existing narratives of user-led and open-source innovation. They also call for more exploration of what it actually means to be locally involved in this kind of technologically mediated and global form of patient engagement. Building on empirical research conducted in the German healthcare context, we explore the different forms of material participation encountered among a group of people with T1D (who describe themselves as loopers), who are engaged in the development and usage of this open-source technology. Introducing the concept of device activism, we retrace three different device-centered narratives that show how a globally shared concern and political participation through technology use varies with local practices. Hereby we stress that the engagement in the #WeAreNotWaiting movement is both shaped by and is shaping the matters of concerns: devices in, on, and with bodies.

## Introduction

In different contexts scholars in Sociology and Science and Technologies Studies (STS) showed how people affected by diseases organize themselves and advocate for their needs, such as for example the HIV/AIDS epidemic (Epstein 1995, 1998; Moletsane and Lesko 2004; Mbali 2005; Wilson et al. 2017), muscular dystrophy (Rabeharisoa 2006) or breast cancer (Klawiter, 1999; King 2004; Cheded and Hopkinson 2021). Such practices challenge traditional conceptions of medical authority and blur the boundaries of science and society by changing common self-description of patienthood and epistemologies of health and illness (Brown et al. 2004; Wehling et al. 2015; Geiger 2021). Over time, the focus of patient activists has shifted from solely advocating for patients’ rights and interests to actively intervene in biomedical research, doing “research in the wild” (Callon and Rabeharisoa 2003), with the goal of bringing attention to “undone science” (Frickel et al. 2010; Hess 2016). Rabeharisoa and colleagues have referred to this as “evidence-based activism” (Rabeharisoa et al. 2014) and pointed out how this form of activism changes the “distribution of competencies and prerogatives between patients and specialists” (Rabeharisoa and Doganova 2021, p. 64).

With recent shifts in healthcare towards technologization, digitization, and personalization of healthcare, there is yet another shift to observe in patient activism. Increasingly, ‘patients’^1^ not only advocate for or engage with researchers and engineers to change what is known and can be done about their condition. They take the means of production into their own hands and create the advocated change themselves through user/patient-led processes of technological innovation (Gallegos et al. 2018; Murray 2020).

In our article, we focus on the latter form of patient activism in the context of type 1 diabetes (T1D): Here, contemporary self-care regimes are built around the expectation that people with T1D take over a substantial part of the therapy themselves (Jansky 2021; Danesi et al. 2020; Piras and Miele 2017; Kingod 2018). T1D is a chronic disease in which the pancreas is not producing insulin and the hormone has to be administered exogenously. This leads to the expectation that people with T1D need to “think like a pancreas” (Plotnick and Henderson, 1998). Thus, they have to take over the tasks of the organ by using pharmaceutical and technological aids. A wide range of technical devices enables the individual manual measurement of blood glucose levels, as well as the administration of insulin (Kesavadev et al. 2020a; Liggins 2020; Mol 2009; Mol and Law 2004). In the German standard therapy, where this study is situated, people with T1D are commonly interacting with at least two devices in their daily self-care practices: continuous glucose monitoring (CGM) devices and insulin pumps. Within this form of self-care, bodies and devices are entangled, forming a “symbiotic relationship” (Garfinkel 2021). In the literature the ‘diabetic body’ is therefore often described as a “cyborg body” (Forlano 2016; Hatch et al. 2020). This is a reference to Haraway’s famous notion of Cyborg (1991, p. 150): “a cybernetic organism, a hybrid of machine and organism, a creature of social reality as well as a creature of fiction.”

Thus, on the one hand, device and user are intimately entangled (Forlano 2016), in the use of the devices. On the other hand, individuals with T1D are confronted with hardly any transparency regarding the use of the health data these medical devices create and collect, little interoperability between devices by different manufacturers, and a huge mental burden of repetitive data work (Gottlieb 2021). Individuals with T1D who generated the data have little room for engaging with data and devices beyond being the *user* (Jansky 2021, p. 138). For example, they often do not have full authority to access, view, download and use data as they wish, another example is that they do not feel properly informed where the servers are located that store their data. In an autoethnographic account on living as an individual with T1D, Forlano (2016) points an important example. In her case, two popular device manufacturers did not offer software to read the generated data on all operating systems. Another well-known issue is the alarm settings of insulin pumps, which operate with standard values often not in sync with the individual bodies and lives of users (Lewis 2019).

In the early 2010s data-driven (health) activism increasingly occurred (Kish and Topol 2015; Lehtiniemi and Ruckenstein 2019). In the same period people with T1D started to share the issues described in the previous paragraph in their technologized self-care routines using the Twitter hashtag #WeAreNotWaiting^2^ (addressed to the device manufacturers). This not only helped to draw the attention of the broader public to this social claim, which might otherwise not have received much recognition^3^ (Zappavigna 2015, p. 274; Williams 2015), it also connected people across the world in their shared concerns.^4^

The (online) critique and protest of the monopolization of (patients’) data by commercial manufacturers over time became a point of departure for patient-initiated innovation (Kesavadev et al. 2020b; Kaziunas et al. 2017; Kaziunas 2018). This started out with Nightscout, an open-source project “to access, view, and interpret data that commercial continuous glucose meters produced but had always been locked behind a black box, unattainable” (Kaziunas et al. 2017, p. 3). Following this open-source strive for data access, a growing community of people with T1D and carers started to reverse-engineer the commercial devices. Furthermore, the community created an open-source closed-loop system that would automate the repetitive tasks in their self-care making the instructions ‘free’ and ‘open’ for anyone to use, modify and share (Lewis and Leibrand 2016; Lewis 2018).^5^ In practice this means that now an algorithm would take over the practice of “doing pancreas” (Wiedeman 2016): instead of looking at the glucose values that the CGM were measuring and then accordingly administer insulin, the algorithm would be inserted as the communication vehicle for these two devices. This would relieve the user of the burden of constant decision-making about insulin dosages, as this would be “primarily undertaken by the algorithm rather than the user” (Cleal et al. 2021, p. 3). Although engaging in this practice demands a lot of determination and mobilization of social and material resources, closing the loop advantages both clinical outcomes (Braune et al. 2021), as well as the quality of life (Schipp et al. 2021).

Loopers (which is how the members of the movement describe themselves) might also be doing “research in the wild” (Callon and Rabeharisoa 2003), however, their goal is not (only) to draw the attention of the biomedical sphere to this undone science: they (mainly) channel their critique of the current state of the device-industry to create, as innovation studies scholars Demonaco et al. (2019, p. 82) point out, “solutions for themselves”.

This poses new questions concerning the engagement of affected persons not only regarding their care, but also in shaping the epistemological, political, and technological conditions for their treatment.

The characteristics of the #WeAreNotWaiting movement as a digitally enabled and technologically engaged global activist patient collective feed into existing narratives of user-led and open-source innovation (von Hippel 2006, 2009, 2016). In the innovation studies literature looping has already been framed as the example par excellence of user-driven, and democratized innovation (Demonaco et al. 2019; Demonaco and von Hippel 2019). However, these celebratory characterizations of a global community that took matters into their own hands offer little insight into the actual engagement and practices of the individuals involved.

Consequently, they also deflect from the local and material situatedness of such practices in their national healthcare, i.e., sociopolitical contexts such as healthcare and public (health) policies. Both in public discourse and in the academic literature, the *global* characteristics of this activism are in focus. However, the engagement in this movement is also always a local, individual, embodied, and intimate practice with devices. To grasp this observed entanglement of global and local, intimate and public practices of activism, we will utilize the term *glocal* (Swyngedouw 1997; Escobar 2001; Polk 2014; Forno and Graziano 2018).^6^

Against this background, the aim of this article is to understand the diverse and locally situated forms of collective and political agency that materialize when persons with T1D in a collaborative way develop and apply these “actually existing alternatives” (Kelty 2008, p. 3) to the standard self-care regime devices. We suggest the concept of *device activism*, to understand how this engagement is shaped by and is shaping devices. Device activism is a form of collective engagement in which devices are central “matters of concern” (Latour 2004). Our proposed concept of device activism aims to capture the varied forms of (political) engagement among patients and patient groups concerning devices in, on and with bodies. In this form of activism, devices are not only the aim of activism, they are also its medium. For “practical cyborgs with T1D” (Garfinkel 2021), devices, such as insulin pumps and CGMs play a significant role in everyday life. With device activism, loopers thus take these disease and life defining devices and use them to make change to and express criticism of manufactures and standard self-care regimes.

With the concept of device activism at hand, and building on empirical research, we provide a new perspective on different forms of global and online participation and health activism. In developing this novel perspective, an important aim is to not lose sight of the material and local dimensions and variations. Living with and depending on an embodied intimate relationship with devices is the central point of this observed activism. This should not be overlooked by only focusing on the ‘bigger picture’ of a global activist community. As more and more personal medical devices (Farrington 2017) enter our lives, we foresee a need to understand new forms of device-related engagement. Empirically, we draw upon ethnographic research conducted in the context of the German #WeAreNotWaiting movement.

In what follows, we first offer a short overview of the different strands of literature that we use as sources of inspiration for the concept of device activism. We then lay out the empirical setting and the methodology, before we introduce our empirical analyses. Finally, we discuss how device activism as a concept may contribute to future research on health movements.

## Theoretical framework

### The recursiveness of participation

In the sociological and anthropological literature on health activism, different notions have been used to signify the collective in question—groups, communities, movements^7^—as have different aspects of their engagement—“embodied” (Brown et al. 2004), “evidence-based” (Rabeharisoa et al. 2014), “corporatist” or “confrontational” (O’Donovan 2007).

What makes the phenomenon of #WeAreNotWaiting distinct, within this context, is that code, data, and (digital) devices are their shared concern and the means through which loopers engage individually and collectively. The closed-loop that they build between glucose monitoring device and insulin pump is mirrored in the ‘loop’ of community members, and the code and resources made available within the online open-source communities. Because such recursiveness is at the core of the looper community, we build on Christoph Kelty’s (2008) concept of *recursive public*s. Kelty is inspired by the mathematical concept of recursive functions, which in programming call on themselves during execution. In his ethnographic exploration of open-source practices, the author describes how the open-source sphere “is constituted by a shared concern for maintaining the means of associating through which they come together as a public” (Kelty 2008, p. 100). An outstanding characteristic of open-source software is “a self-determining, collective, politically independent mode of creating very complex technical objects that are made publicly and freely available to everyone” (Kelty 2008, p. 11). The recursive public is not bound to geographical regions and unfolds online. As we pointed out previously, the #WeAreNotWaiting movement developed in the light of increasing data activism in healthcare. Within in this context, free, and open-source software is intended to be the opposite of what the biomedical and technological manufacturers are criticized for: open-source code as open and transparent, viewed and modified by anyone, evolving as a collaborative effort (DeLanda 2001, Par. 1). Kelty (2013) describes how the open-source community is not about creating something static, but about something that is constantly becoming. This is important for understanding the #WeAreNotWaiting movement as a recursive public: it is about possibilities to think and create differently regarding personal healthcare and somatic issues.

### Material participation in healthcare

While the concept of the recursive public gives us a good tool to describe the ‘looping’ nature of the engagement in the #WeAreNotWaiting movement, the issues of concern for the recursive public of loopers are not only the open-source technology. The most prominent issue is *chronic living* (Wahlberg et al. 2021). Theories of embodied health movements pointed out how these movements are making the body and the embodied experiences of people with diseases central, hence giving “people with the disease or condition a lived perspective that is unavailable to others” (Brown et al. 2004, p. 56). Accordingly, in a more and more technologically situated healthcare setting, the materiality of technological devices, and the entanglement of bodies, devices and data needs to be systematically addressed. “[T]he distinct everyday rituals and embodied experiences of living with particular kinds of embedded devices and materials” (Forlano 2016, p. 7) need to be taking into account when looking at health movements in technologized and digitized healthcare settings. Things such as “[c]lear plastic tubes poking just beneath the skin’s surface and small grey radio transmitters protruding outwards in unfortunate bumps” (Forlano 2016) are part of the lived experiences of people with T1D and are an essential parts of the engagement and shared matter of concern in the #WeAreNotWaiting movement.

In order to better grasp the material dimension of the *engagement* in the #WeAreNotwaiting movement, we use the notion of “tactic of material participation” (Nielsen and Langstrup 2018). Inspired by the work of Marres (2012), this notion can help us understand how users of digital health technologies are engaged in both individual intimate care management and in health innovation and thus participate in normatively varied ways through tools, technologies, and devices (Nielsen and Langstrup 2018, p. 264). Originally, her concept of “material participation” (Marres 2012) entailed how everyday material entities and environments “have distinctive capacities to engage people'' (Marres 2012, p. 1). Furthermore, it included how the expectations of users participating in a political way are shaped by specific material formations. Nielsen and Langstrup (2018) added the concept of “tactics” with reference to de Certeau (de Certeau, 1984; see also Sharon 2015). The authors pointed out that people, throughout the mundane everyday practices, at the same time meet these participatory expectations, but in in highly creative and varied ways, using different tactics. In our approach, “tactics of material participation” (Nielsen and Langstrup 2018) help us to see that the engagement within the #WeAreNotWaiting movement is not an abstract issue. Rather, the entanglement of health-related engagement, bodily practices and glocal activism are plural reactions of people to devices that are essential for their chronic living (Wahlberg et al. 2021). Moreover, it helps us register the actual variability of the local forms of engagement, rather than expecting that every user of an open-source closed-loop system does so in similar ways and with similar intentions.

### Device activism

Before we venture into the analysis of our empirical material, we summarize the different strands of literature, which have laid the ground for thinking about what device activism can entail.

Firstly, we need to acknowledge the recursiveness of the engagement. We define device activism as a recursive practice, allowing us to describe how looping can be understood as a “certain immanent critique” (Kelty 2005, p. 186) of the existing politics of chronic living in diabetes. Importantly, and this is our second characteristic of device activism—the engagement is fundamentally material. It is the bodies, the devices in, on and with bodies that make up chronic living and this is the shared concern. Finally, device activism further involves varying versions of participation (Marres 2012; Nielsen and Langstrup 2018). Regardless of the specific motivations people start to get interested in the #WeAreNotWaiting movement and open-source closed-loop technologies, as soon as they interact with the technologies and the embedded devices, their actions are also political, but this political participation materializes in highly creative and diverse ways.

For our following empirical analysis, the conceptualization of device activism enables us to understand this form of political engagement in its different dimensions: from global hashtag activism to local, embodied critique of current healthcare politics.

### Setting and methods

Empirically, our article zooms in and out of the German healthcare context. The individuals we follow in our empirical investigation are situated in the German healthcare context. Germany is a wealthy western European country with universal healthcare and rather conservative approaches of adopting digital health technologies (BertelsmannStiftung 2019). But, importantly, the individuals engage in and with a global movement, that cannot easily be placed within “national containers’’ (Beck 2007). As Kingod (2018, p. 4) points out, to understand contemporary patienthood, one has to follow people through their online and offline worlds. To take this into account and grasp the entangled local and global (the glocal) dimensions of our researched phenomenon, we, took inspiration from Ayo Wahlberg (2018)’s idea of an assemblage ethnography. In order to situate the analysis deeper on an individual, collective, material, and discursive level, we further followed a situational analysis approach as suggested by Clarke (2005; Clarke et al. 2015). With the perception that “[t]here is no such thing as ‘context'’” (Clarke et al. 2015, p. 98), the different elements of the researched situation are seen as constitutive parts of it and not solely as the surrounding of a researched phenomenon. This helps to understand both the ‘bigger picture’ of the global movement and the intimate, local practices of engagement. Furthermore, as our focus lies within the material, the devices, the situational analysis approach allows us to “specify the nonhuman elements in the situation” (Clarke et al. 2015, p. 101).

In the fieldwork, the first author followed different actors and stories (Latour 2005, p. 12), rather than focusing on one specific site (Marcus 1995). To get a better understanding of public and broader negotiations, we triangulated heterogeneous data sources and included media reports, blog posts and statements of regulatory bodies into the analysis. The first author conducted 28 problem-centered in-depth interviews (Reiter and Witzel 2019) with loopers, ex-loopers, their relatives, and healthcare professionals. The interview participants were recruited through meet-up groups, the snowball method and two participants were recruited through their public diabetes blogs. Some of them were active on Twitter and other social media platforms and thus engaged in the hashtag activism around the #WeAreNotWaiting hashtag. Some were not active on social media at all and were only participating at local meetups.

The interviews lasted from approximately 20 min to two hours. Out of the interviewed participants, twelve were women, sixteen men. The age ranged from 22 to 81. Six participants were healthcare professionals, of which two were people without T1D. The interviews were conducted in German. There were two exceptions in which the interviews were conducted in English. Quoted passages were translated in collaboration with a bilingual native speaker. The interviews were recorded and later transcribed verbatim. As the German community consists of a small group and some people in the German community are well-known on social media, the gender of participants is sometimes changed in the quoted passages, and different pseudonyms for the same person are used to protect their anonymity (Saunders et al. 2014). Data analysis and gathering were mutually informed by one another, following an iterative logic. Data collected early on in this analysis served as a starting point, to direct the strategy of theoretical sampling (Clarke et al. 2015, p. 101f.). For the analysis, we used a constructive grounded theory approach (Charmaz 2000), and we further followed the mapping strategies of the situational analysis (Clarke 2005; Clarke et al. 2015), to get a better “theoretical grasp of the phenomenon” (Clarke et al. 2015, p. 108).

## Findings: device-centered narratives of activism

### #WeAreNotWaiting and the OPEN Project

The #WeAreNotWaiting movement is often referred to as a global movement; the focus here shifts from the individual experiences to the “virtual collaboration of a global community” (Lee et al. 2016, p. 1447). Public debates about the practice of looping and the #WeAreNotWaiting movement are concerned with this community, as one unity, and also a few individuals who have a strong voice in public discourse, such as for example Dana Lewis, who can be described as one of the ‘pioneers’ of looping. She and her now-husband first shared the instructions for the closed-loop system open-source and by doing so are credited with initiating this global community (Lewis, 2019). Some of the people encountered in the empirical work of the first author could be placed in a similar public position in the German context. Kim, a healthcare professional, with a rather strong public voice in the German debate, explains what this means as follows:

I try to use my exposed position to fight for us. I don't know, there is no German word for 'advocacy' but to fight for our rights and to push for our interests and this always needs some kind of spokesperson. I am maybe the Greta Thunberg of medical devices (both laugh) and I like doing that and I just try to highlight what others have already researched and what others have already said [...]. I just try to get our message out to the right audience as much as possible.

Kim uses their public position, which came with them^8^ being a healthcare professional and a very vocal not only advocate but a member of the #WeAreNotWaiting movement, to bring to the public what the community demands, and thus acts as a sort of spokesperson, in the German context and beyond that. Kim knows the biomedical discourse around closed-loop systems and is familiar with the traditional research sector and device industry, and at the same time has the shared “experiential knowledge” (Borkman 1976) of living with the chronic disease and can claim epistemic authority by relating and contrasting these to professional expertise also accessing more formal, established research areas (Epstein 1995; Callard and Perego 2021).

The strive for partaking in the traditional research sector and thus further validate the work of the #WeAreNotWaiting movement can be understood best when looking at the OPEN project:

The “OPEN” project brings together an international and intersectoral consortium of patient innovators, clinicians, social scientists, computer scientists, and patient advocacy organizations in order to investigate various aspects of Do-it-Yourself Artificial Pancreas Systems (DIYAPS) that are used by an increasing number of people with diabetes. (https://open-diabetes.eu/en/welcome/)

Seeing the growing interest in their activities, the community organized itself, and the OPEN project was established. An EU-funded research project where ‘traditional’ researchers work together with individuals who do not necessarily have academic credentials. The majority of the ‘traditional’ researchers are also part of the looper community. At the same time other researchers—like the second author of this paper—were invited to participate due to specific expertise needed for the project. It is emphasized that while this research project is situated in the university research structures and funded by prominent European research funds, at the core it is ‘of the community for the community’. Most members of the research consortium explain in their research profiles, how they personally relate to the cause of the #WeAreNotWaiting movement. Other loopers are kept in the loop by receiving regular newsletters and information via Facebook groups. The project was initiated during the fieldwork time of the first author, who is not involved in the OPEN consortium, and on more than one occasion informants would not only refer to the OPEN project but would emphasize their participation in the studies of the OPEN project. The OPEN project is an example of how the community strives for epistemic legitimacy and acceptance from the established actors in biomedical research and development. The initiators of the OPEN project use the funding for establishing a research project, where the affected are themselves, project leads and not ‘just’ co-researchers, as it would be for example in citizen science (Fiske et al. 2019; Prainsack 2017). Within the OPEN project ‘patients’ are in the unique situation that they are more than “epistemic factors” (Falke 2018), and their knowledge is regarded as especially valuable from device manufacturers, research and development institutions. The OPEN project exemplifies how the community around the #WeAreNotWaiting movement aims to establish epistemic legitimacy in a global sense and to push for a globalized critique of innovation politics and practices in contemporary biomedical settings. As such, the observed practices fit into what Rabeharisoa et al. (2014) describe as “evidence-based activism”: Within this type of activism, people are interested in collectively producing and mobilizing knowledge, referred to by Akrich et al. (2012, p. 31) as “evidential work”. They formulate political claims addressed at both global and local actors in the healthcare sphere.

The #WeAreNotWaiting movement can be understood as a quest for a “technical cure” (Heinemann 2017) for T1D. Loopers take means of production into their own hands and thus challenge production and knowledge practices in biomedicine. The #WeAreNotWaiting movement did not emerge in a vacuum, it builds on these forms of patient activism. However, if we look at the #WeAreNotWaiting movement only through the above-mentioned theoretical lenses and narratives focusing on its ‘global’ characteristics, we neglect central aspects of the material and local ways of participation and engagement in this movement where the entwinement of the global, the local and the embodied participation is even more clear.

### Uninvited material participation

The table of the regular looper meet-up group is always easy to spot in the small restaurant. It’s November and during the week, so there is no one except the looper meetup group in this small restaurant that is part of an allotment garden colony a bit outside of the city. At two tables people of different age groups and genders sit together, there is a high level of noise, as everyone is chatting, some people are already eating, some are just drinking, and others go around the tables or chat in smaller groups. What makes this situation particular, is the variety of different technical devices on the table and people sit at the table with several devices in their hands: People mostly have two smartphones placed in front of them at the table, they wear a glucose sensor visible on their bodies, a few insulin pumps are out on the table, catheter and injection areas on the skin sometimes apparent, sometimes not, and the smartwatch presence is higher than elsewhere. There is always at least one person with their laptop out. And then there is Marcel. He is an electro engineer, and he has access to a 3D printer. This time, he printed patches to use over the transmitter for the sensor, so it won’t fall off easily: it even comes in different colors, and he has brought at least 10 patches and is giving them away to the members of the meetup group. Mara says she had been looking forward to this, takes a patch, exposes her upper arm, reveals a sensor on her skin and holds the patch over the sensor to show us what it would look like. (Summary of a field note)

The contrast between the above-described situation in the small restaurant and the before-mentioned OPEN project shows how multifaceted the engagement can be. The OPEN Project strives to generate scientific evidence for the closed-loop systems’ effectiveness on a global scale. People that use the systems in their everyday lives, without explicitly aiming at changing anything, but their own treatment, are still through their engagement with devices participating in a politically significant way. With the widespread “participatory turn in healthcare” (Prainsack 2011, 2014) patients broadly and people with T1D extensively are “invited to participate” in their own treatment and through their material devices (Nielsen and Langstrup 2018). Crucially, when people with T1D are participating in the #WeAreNotWaiting movement, their engagement can be characterized as ‘uninvited material participation’: they circumvent the original invitation/inscription of devices and share the devices and instructions, to engage them in different ways than intended by the manufacturers. In the context of the #WeAreNotWaiting movement, devices are not just tinkered with, they are repurposed: some are not even intended as medical devices, and some devices are created by the people in the community to fit their immediate purpose. The devices change their properties by being mediated by an open-source control algorithm—and with that they do become invited to participate anew. Loopers engage in disrupting distinctions of what is considered medical devices (such as insulin pumps or CGM), mundane everyday life devices (such as smartphones or smartwatches), user and developer, and activist and a person who just wants to use a better tool for their self-care. Devices—their materiality, their connectivity, their intimate entanglement with bodies and lives, and their politics—are at the center of the looping community and the entry point for uninvited participation. For some, having a specific device can be the gateway to engagement. Franz, a carpenter in his late thirties, for example, retraces how he first encountered the closed-loop technology:

It was actually a coincidence that I was prescribed the right pump. And that this step towards this first loop attempt was actually a very small one because I didn't actually need any new hardware. I have the Dana pump and it literally invited me to try out the loop.

Franz’s narrative of how he started out with looping, is very much device-centered: he was recounting how he was “prescribed the right pump” to loop. He further prescribes the device agency by explaining that it was the pump that invited him to try out looping. For people without the “right pump”, the hunt for these specific devices is what is a center of their narratives of how they started out looping. Sabrina explains how she got her pump from someone at a local meet-up:

And the woman says: "Yes, I still have four old Medtronic pumps at home. If you drive me home right after dinner, I'll give you one." I was like, "Deal." I was mega excited and so I drove her home and I was like: Oh my God, I'll give you money for it. She was like, "No, for God's sake!" I felt really bad because I just knew how expensive these pumps are now. And I was like, "But please, they're really expensive. I can pay you for them." And she really said: "Wow, please just take it, because it's just been lying around here for years". […] I was so excited on the drive because I thought: Yes, now I can loop. I can still remember it, even if I can't really understand now why I was so incredibly excited. But then, on that Thursday evening, I got straight to the computer and started downloading the first things.

Asked how she ventured into looping; Sabrina’s narrative is similar to Franz’s device centered. With the help of another person with T1D and the social gathering of a local meet-up, she was able to gather the ‘ingredients’ to set up the loop. This pay-it-forward ethos is a recurring theme in the looper community. It is also interesting in relation to the notion of the ‘cyborg diabetic body’, where the symbiotic relationship with devices, in, on and with bodies be dissolved and then re-entered with the device that was once part of another person's ‘cyborg body’.

In different instances, the first author observed people sharing their devices. Sometimes this happens, similar to Sabrina's story, just by chance, other times it is more planed: The first author once participated at a meet-up, and at the beginning of the gathering the hosts pointed out a big bin, in which everyone could put devices they no longer needed, which could then be passed on to others, who need them. While these practices illustrate the centrality of devices, they also point to a recursiveness of the community, where access to the means of participation is a shared concern, overriding any concern for ownership or market value (the devices are very expensive) and any duty toward individuals or institutions outside the community (such as healthcare providers or welfare services).

Sometimes gathering all the different devices is a difficult undertaking and one often needs others to help. The significance of certain devices is so large that there have even been black markets on eBay for the special pumps (Zhang 2019) and at looper meetings, it is common for the devices to be exchanged ‘under the table’, similar to what Sabrina explains. These physical meet-ups are another way how people with T1D are venturing into engaging in the #WeAreNotWaiting movement. People with T1D are often encouraged to participate at T1D meet-ups and engage in T1D online peer-to-peer support groups from their healthcare professionals, as the support groups are seen as an “additional support structure” (Crocket 2019). These structures (where people with T1D *are* in fact invited to participate by formal health authorities)—both online and offline—often offer a way to venture into engaging in the #WeAreNotWaiting movement—into *uninvited* participation. While materially and collectively invited, it is not as prescribed by the original device inscription nor the formal healthcare authorities. Sandra, a legal secretary in her forties, retraces how she encountered looping while she was on a large online diabetes support platform:

[…] and in the context of getting the pump, I became more active in the internet community. […] I got my first information about the pump and also the CGM there, and then I came into contact with other people who had a similar attitude to life as me. I think it was about 6 or 7 people who talked about everything on this platform and one person suddenly started to share what he had discovered on the Internet: the possibility of looping […]. I'm interested in technology, yes, but I'm not tech-savvy. So first, I was just reading along with my eyes wide open what looping was all about and what you can do with it and what you can build and solder and do and I always just thought, “Wow, that would be cool! I'd love to do that but shit you can't do it yourself” and then I actually followed quite attentively the whole time how first this one person and then shortly afterward the next person started to build themselves a loop.

For Sandra, again the device was the central point of departure, with her aiming for getting more information about the new device in her care routine. She connects, as many individuals with T1D (Kingod 2018), with others online, and this is where she first learns about looping. Her unfolding narrative took place in 2015, a period in which there were few loopers in Germany.

With retracing the different meeting spaces (online and offline) we can see how multifaceted this movement is and how it is at the same time very global and international and then very local and regional. While the hashtag is important for the international connection and the development of this open-source technology, on a more individual and local level the social, geographical, and material aspects might be the entry point to what we refer to as ‘uninvited material participation’. This perfectly illustrates the glocal nature of the movement. Xaver, a businessman in his 70s explains how the looper meet-up that he attends started as a very small group of patients meeting to discuss new diabetes technologies in the office of their diabetologist:

Yes, I don't know exactly when the first one was. I think it was with the physician where some of us are, the one in [city part in a larger city in Germany], and with whom we met at the beginning - he always did his training in his doctor’s office. And we met there at the beginning, I think the worries from all sides were a bit bigger, and we were a bit more cautious, but then we met there and at some point, the room became too small, I guess it was in- I would say in the spring [of 2017], and then first there in the doctor's office and then at some point in the summer we moved to the [new location] and then we became more and more people, and the second to last time I think there were about 50 to 60 //wow, amazing// people there.

Xaver’s narration from how his local looper meet-up started and changed, is especially interesting, as it illustrates quite well that the engagement that we observe has concrete local consequences. In no word did Xaver even mention the online community in his statement. He later explains that while he reads the online documentation, he does not participate in any online discussions. Still, he is one of the most senior regulars at the local meet-up. Most of the people that the first author interviewed and talked to explained that even when they first just read online about the technology and had to set up the system themselves, at some point they felt the need to exchange with others face to face. It was also mentioned multiple times that it can be easier to engage in peer-to-peer support for newer members when one is physically in the same space, as looping is often characterized by readjusting and tinkering with devices and bodies.

To summarize, the engagement in the #WeAreNotWaiting movement can only be understood if we understand it in its materiality and the centrality of the devices in this situation. The device acts here as an entry point to being engaged. While research on embodied health movement already points out how important the embodied experiences of having a disease are, to fully understand it, here the materiality of devices on, in, and with bodies and the entanglement of bodies and technology is at the center of experiences. In order to engage in the #WeAreNotWaiting movement, one needs to recognize the role of what is often described in the empirical material as “hardware”: an insulin pump, a CGM, and a central controlling device (mostly a smartphone). This centrality of the devices is woven into all the different tactics of material participation and is the basis for the engagement. The example of peer-to-peer support and meet-ups can be used to empirically retrace what we are referring to as uninvited material participation. people with T1D initiate and participate in local meetups, that might not be strongly connected to the bigger global discourses around the #WeAreNotWaitng movement. Nevertheless, they are entangled within the global movement. The act of engaging with devices in ways that were not intended by the manufacturer—the reverse engineering- can be interpreted as a form of political participation. While loopers such as Sabrina, Franz, or Xaver are predominantly engaging locally, and are focused on their own individual care, they share the same, or very similar, concerns as the ‘globally’ engaged loopers. Importantly, as mentioned before, we want to point out that while this form of engagement is ‘uninvited’ in the sense, that it was not what manufacturers had in mind when designing the devices, the engagement is ‘invited’ by other loopers. On top of that manufacturers and activists are not always necessarily on opposite sides, as there are now increasingly projects were loopers are also commercializing their innovations or work together with the commercial manufacturers (see for example: Tidepool or Bigfoot Medical). Our empirical analysis provides insights into the global market of these innovations and how activists are collaborating with the industry. Having said that, this is not the main contribution of this article, here more empirical research is needed.

### Localizing activism in national healthcare contexts

Even though the individuals engaging in the #WeAreNotWaiting might have different goals, they are however usually part of a national healthcare system that shapes their experiences. This became for example evident when the discussion of the legality of the closed-loop system in the German context were heated during the time of the first author’s empirical research. These concerns were prompted at two public events: firstly the statement of the German diabetology association in 2018, and secondly an article in a patient information journal from a lawyer, pointing out different possible legal consequences of looping. This put loopers in the spotlight of public debate in the healthcare sphere in Germany, while at the same time bringing them in an even more precarious situation. Alios responded to this issue by explaining how an official organization within in the German healthcare context could be useful:

But in a community where there is no organizational structure in Germany, an association or a self-help group, there is also no one who can legally defend themselves, which I think is a huge problem, but let's see if that changes in the future. So that's something I don't like in the community at the moment, that there are no official structures, no official voice [...]. Open source and do-it-yourself is basically everybody does it for themselves or everybody does it together and everybody does a part of it. Everybody is a part of it but who is behind it or who is in charge of it? […] At the moment it's just one person who says "yes, I speak for or against this in my own name" but it's probably always better if you can say "I'm a patient association or a self-help group that has so and so many members nationwide and we demand that dot dot dot". That's something different than when a person stands up and says “I'm looping myself and I'm active there and asks you to think about it” that has a completely different effect. That for me is something that should perhaps be considered in the community but of course, not everyone is in favor of it and there are arguments for and against it.

Alios explains how it would be helpful to have some sort of ‘patient association’ or another form of organization that could stand up for the community. For Alios, being more organized on a national level would help in situations like that, as people would not only speak for themselves but as legitimate representatives of the community. This strive for more organization contrasts with the open-source ideas that the #WeAreNotWaiting movement is oriented at. But on a national level, and considering the public discussions around the legality of the open-source closed-loop system, it made sense for Alios to think of an organization that could be a representative of the community’s needs. People in the German looper community orient themselves at the issues of the global community, they are however also intertwined with their local healthcare context. As a consequence, questions that might not be very relevant for the, for-example, US community could be very important to people looping in Germany. In other words, engaging in this global movement has local everyday life consequences.

This can also be well retracted by looking at the issue of getting access to the “right” devices. Not every insulin pump or CGM is suitable for looping, so people refer to the devices that are compatible for the open-source endeavor as “right” devices (see in our article Franz’s quote). In countries such as for example Australia (Schipp et al. 2021) people can have financial struggles to buy the “right” devices and pay for the running costs as they are not reimbursed or subsidized (ibid). Within the German healthcare setting, the access to the devices is not mediated by financial issues, since the devices are reimbursed by healthcare insurances. From there, it follows that in discussions about the access to the “right” devices, public healthcare insurances are considered as actors in the situation. The conversation in the field often moved in the direction of how to convince the health insurance that one needs a certain device, or how to conceal to the health insurer that one is looping. Even though German healthcare insurances take over the costs of most of the devices that one needs to loop, changing the technological system you are using entails a difficult and bureaucratic process. Nina explains how the German community is dealing with these issues:

So sometimes there are systems that come from the health insurance company and that are then sold to others. […] But then, what is also interesting, for example, there is this Dexcom, you probably already heard, Dexcom G6. And there are these transmitters for it. And they last three or four months, there's a battery inside. Then there are apps that you can use because it has a stop in it. The battery would actually continue to run. But it stops after three months because they [the manufacturer] actually want to earn something from it. […] And then people said: “Okay, let's build an app so that you can extend it.“ So now you can screw these G6s open so that you can charge the transmitters. So, they built a charging device into it and took the battery out. And then they modified it. And of course, that's what's sold internally (in the community). Because you have to know how to help yourself somehow because you don't get these sensors from the health insurance company. They are so expensive. And I mean, I've already reached the point where I can't get the Dexcom G6 approved either, I'm fighting for it and my health insurance only pays for the other system, which I don't want, which someone else wants. But it happens automatically. Then someone says, “Okay, come here, I'll give you this one, you give me so and so much of this one, so it roughly balances out.“ But then also really these transmitters that are built. You buy those, of course. Honestly, if I saw that one on eBay, I'd buy it. Or anywhere. And I wouldn't question who was selling it.

For the closed-loop set up one needs specific devices and the question of how to get access to these devices is intertwined with local national healthcare regulations. Nina describes here how her healthcare insurance would not approve her a Dexcom G6, which is a continuous glucose monitoring device that, at the time of the interview (2019), was especially popular in the US. In Germany Abbott’s Freestyle Libre was more common to be approved by health insurances. The devices that are at the center of the different narratives of activism that we retrace are still bound to the specificities of the local context: they are medical devices that are governed by regulatory bodies and national healthcare systems. In the German #WeAreNotWaiting context, the negotiation of what public health insurers think of the closed-loop systems and the engagement in the community are very important, whereas in the global online community they hardly seem to play a role at all. Notably, Nina stating that she would not even “question who was selling” the device to her also points out how the local involvement is also always just one step away from being transgressed by the global. To get the “right” device, the local politics of a national health system are dismissed for the global, online, commercial market, such as eBay. Nevertheless, when we only look at the global community, we glance over that engaging in the community is very different for people and it also varies in different healthcare systems.

The two topics that Nina and Alios refer to in their interviews are good examples to retrace how the engagement in this global community is bound—through the importance of the devices—to the national and local healthcare context. It however also challenges these national healthcare structures: As we can illustrate these experiences of lack of support at a local healthcare level actually reinforce a ‘globalization’ and ‘marketization’ of healthcare. The engagement in the #WeAreNotWaitng movement also disrupts or circumvents local health politics. The #WeAreNotWaiting movement, or healthcare movements in general in a digitized healthcare sphere, can neither be only understood in a local way nor only as a global issue.

## Discussion

In our article, we turned our gaze at the #WeAreNotWaiting movement—a case of glocal patient activism that has arisen with an increase in digitalization, technologization, and personalization of healthcare contexts. With its strong connections to the Internet, interconnected personal medical devices, and self-care regimes inviting patients to participate, it connects different forms of (political) engagement that were so far not addressed in the social study of health and patient movements. We suggest that the concept of *device activism* may best capture such collective engagement, where devices are central “matters of concern” (Latour 2004). With this concept, we hope to offer a way to analyze different forms of participation in global and online forms of activism, while not losing sight of the intimate, material, and local dimensions. The #WeAreNotWaiting movement is a prominent case in point, in that it is both shaped by and is shaping these “matters of concern” in very distinct ways: devices in, on, and with bodies. If we only focus on the global activist online community and understand them as a unit, we fail to see the nuanced material dimensions of participation. It is, we have argued, important to not oversee the more subtle forms of engaging in favor of the ‘big’ story of the political economy of health innovation on a global level. The rich literature on health movements and patient activism in Sociology and STS has shown us that advocating for one’s needs and interests in healthcare contexts are diverse forms of political participation. Using and rethinking technological devices for self-care can be situated in this literature, but, also connects it to other STS literature: Technology use, the different “material tactics of participation” (Nielsen and Langstrup 2018), can be understood as political participation and activism (Gottlieb 2021). Current health movement and patient activism literature is so far focused on how people affected by diseases fight for recognition of a disease (Callard and Perego 2021; Dumit 2006) and engage in so far “undone science” (Frickel et al. 2010; Hess 2016). Loopers are creating their own medical devices and radically changing both their own treatment and challenging the political economy of health device innovation. Loopers do not all engage in the same way in the movement, but they share a set of concerns that connects them, which is in relation to dealing with treating oneself with digital health devices. Loopers, no matter if they engage in the more global movement or just on a local level, abandon the technological system that is used in standard therapy and was provided by (public) healthcare insurances in order to use a new innovation.

The concept of device activism gives us a tool to consider the global and the local levels of the engagement and the engagement of these levels, with the focus on the shared concerns of the looper community: chronic living (Wahlberg et al.^2021^) with devices in, on, and with bodies.

In the empirical material, we can retrace a shared set of concerns that is interwoven in all the narratives of engagement in the #WeAreNotWaiting movement. Everyone engaging in the movement is also part of the shaping of new socialtechnical imaginaries (Jasanoff and Kim 2013) of health, where innovation in healthcare should be radically open. The idea of ‘by the community, for the community’ and the doctrine of ‘pay it forward’ shows how the innovation of the closed-loop system only exists if you make it possible for others and thus points to a recursiveness of the engagement. The #WeAreNotWaiting movement emphasizes both the modifiability and participatory element of innovation. This is distinct from concepts such as “evidence based activism” (Rabeharisoa et al. 2014) or “treatment activists” (Epstein 1995), as it is not about making established actors in the biomedical sphere aware of one’s health needs and trying to participate in research: Engaging in the #WeAreNotWaiting movement has an immediate advantage to one’s self-care.

In our empirical material, we can retrace that while #WeAreNotWaiting transcends national boundaries, it still is entangled with national healthcare regimes, local legal regulations, institutions such as healthcare insurance, and first and foremost the chronic living of people engaged in this movement. The decision to loop thus is a material and situated one, not just an ideologically of believing in patient innovation.

While we used the empirical insights into the #WeAreNotWaiting movement to conceptualize the notion of device activism, this concept can be translated onto other cases of activism in the increasingly digitized, technological, and personalized sphere of healthcare, where familiarity with and availability of personal medical devices may make people weary of waiting for the established actors to catch up with the needs and wishes of those affected. We can see this in cases such as sleep-apnoea (Schultz 2019), period-tracking apps (Hendl et al. 2020); hearing loss aids (O'Kane et al. 2019), or just recently open-source ventilators in the context of the COVID-19 pandemic (Pearce 2020; Richterich 2020). While these are very different cases of healthcare issues, and they might not be as organized as the #WeAreNotWaiting movement, the concept of device activism gives us a prism to look through in order to better understand these forms of health activism where the matter of concern is living better with (the help of) technical devices. How this unfolds more specifically in other areas—whether reverse engineering and open-source have similar roles to play, what forms of collaborative action take place within the context of social media, and which local, material practices unfold—these are all questions for empirical exploration.

Before concluding we want to acknowledge the limitations of our analyses. Firstly, we are focusing on a small group of people in a western European country that, as others already have pointed out, is acting in socio-economically better positioned spaces and conditions (Gottlieb 2021; Gottlieb and Cluck 2019; Hatch et al. 2020). One needs to not only have access to the devices, but also have the time, as well as economic and social resources in order to not wait and engage in device activism. The device activism of the #WeAreNotWaiting movement can be viewed as glocal repair work of inadequate or precarious chronic care infrastructures (Kaziunas et al. 2019). The main difference is that in this case—in contrast to some of the cases described by other researchers (Ibid; Duclos and Sánchez Criado 2020; Sánchez Criado et al. 2015) —the solutions and actors have moved fast from the infrastructural shadows to the innovation limelight. The societal positions of the people engaged within the movement, their individual and collective resources and the movement’s alignment with contemporary imaginaries of digital health futures and (open-source) innovation, which have all played a part in this. While this was not the aim of our article, we want to stress that structural inequalities are weaved in this observed movement and need be thought of when thinking of device activism, here more research is needed. Furthermore, we could not elaborate in detail why people are engaging in the #WeAreNotWaiting movement and how their self-care routines are changing with looping. We refer the reader to existing work (especially the OPEN project) that are looking into the motivations of loopers (Schipp et al. 2021; Braune et al. 2021).

## Conclusion

The idea of *device activism* gives social sciences researchers on patient and health activism and movements a new concept through which to look at patient groups in an area of increasing digitalization and personalization: The recursiveness of these groups and their shared concerns and at the same time the materiality of their engagement which is more and more bound up with personal medical devices and online engagement. It is the social imaginary of controlling the means of their own association and of their own embodied and chronic living which is at the center. The #WeAreNotWaiting movement is a powerful example of a patient movement that is equally focusing on embodiment and recursiveness and might be a herald of what is yet to come in increasingly digitized and individualized healthcare settings. People engaged in the #WeAreNotWaiting movements are not only proposing changes in the future but—through “argument-by-technology” (Kelty 2005, p. 186)—in code—actually making the changes in the present.

## Footnotes

1 From here on out, we will carefully use the term ‘patient’ only in relation to the theoretical concepts that we are building upon, and not to describe the individuals in our researched case. Language matters, and as we will be talking about individuals with a chronic health condition, the term ‘patient’ does not give their experiences of having to live with this disease everyday justice (Schicktanz 2015; Dickinson et al. 2017).

2 #WeAreNotWaiting is not the only hashtag that is utilized, however this one is the one most connected to the looper community and is now movement defining. Other hashtags include: #PayItForward or #NothingAboutUsWithoutUs.

3 This practice is recently also referred to as Hashtag activism (see for example: Williams 2015).

4 There is already a rich literature on how social media is used by patients to connect with each other (see for example: Kingod 2018; Bellander and Lundqvist 2018).

5 The system was first described as “Open artificial pancreas system (OpenAPS)" (Lewis 2019), and the term ‘DIY’ was used as an auxiliary word to describe that the system had to be set up by every individual who wished to use it, as the creators would otherwise be the manufacturers and might be liable for potential faults in the system. The term however sticks much more with the technology and community, than the initially used reference to the open-source nature, and the system is now mostly referred to as DIY Artificial Pancreas System (DIYAPS). The systems “benefit from a fast innovation cycle” (Boughton and Hovorka 2019), as well as transparent, open-source, and customizable algorithms, however, they are not authorized by regulatory bodies.

6 Glocalization gives a term to analyze social movements considering their simultaneous existence on a more global and local context. This term is especially used in the context of environmental movements, as it points out how these movements are both “a defense of local livelihood, culture and environment but it is also a matter of justice claims and global environmental values” (Urkidi and Walter 2011, p. 219). In context of the more recent social media mediated social movements, such as Occupy Wall Street, the Arab spring, the Me Too movement or the Black Lives Matter movement the concept of glocalization can be used to grasp how “online communication tools can serve as a global resource and support structure, from which each initative might pick and choose related to its own needs.” (Polk 2014, p. 139).

7 ‘Movement’ and ‘community’ are both the terms that are most used in the field when describing the collective, we therefore will still use these words to describe the #WeAreNotWaiting movement. The concept ‘recursive public’ is a concept to better understand and describe the dynamic and characteristics of the #WeAreNotWaiting movement. This means we do not see these terms as describing something else, but rather ‘movement’ and ‘community’ as emic terms and ‘recursive public’ as a theoretical concept.

8 In order to ensure the anonymity of the participant, we use they/them pronouns.

## References

[CR1] Akrich, M., M. Leane, C. Roberts, and J. Nunes. 2012. Childbirth activism as evidence-based activism. https://halshs.archives-ouvertes.fr/halshs-00702075/document. Accessed 01 December 2021.

[CR2] Beck U (2007). The cosmopolitan condition: Why methodological nationalism fails. Theory, Culture & Society.

[CR3] Bellander T, Landqvist M (2018). Becoming the expert constructing health knowledge in epistemic communities online. Information, Communication & Society.

[CR4] BertelsmannStiftung. 2019. #SmartHealthSystems. International comparison of digital strategies.

[CR5] Borkman T (1976). Experiential knowledge: A new concept for the analysis of self-help groups. The Social Service Review.

[CR6] Boughton CK, Hovorka R (2019). Is an artificial pancreas (closed-loop system) for type 1 diabetes effective?. Diabetic Medicine.

[CR7] Braune K, Gajewska K, Thieffry A, Lewis DM, Froment T, Odonnell S, Speight J, Hendrieckx C, Schipp J, Skinner T, Langstrup H, Tappe A, Raile K, Cleal B (2021). Why #WeAreNotWaiting-motivations and self-reported outcomes among users of open-source automated insulin delivery systems: multinational survey. Journal for Medidical Internet Research.

[CR8] Brown P, Zavestoski S, Mccormick S, Mayer B, Morello-Frosch R, Gasior Altman R (2004). Embodied health movements: New approaches to social movements in health. Sociology of Health & Illness.

[CR9] Callard F, Perego E (2021). How and why patients made Long Covid. Social Science and Medicine.

[CR10] Callon M, Rabeharisoa V (2003). Research “in the wild” and the shaping of new social identities. Technology in Society.

[CR11] Charmaz K (2000). Constructing grounded theory. a practical guide through qualitative analysis.

[CR12] Cheded M, Hopkinson G, Geiger S (2021). Heroes, villains, and victims: Tracing breast cancer activist movements. Healthcare activism: Markets, morals, and the collective good.

[CR13] Clarke A (2005). Situational Analysis: Grounded Theory After the Postmodern Turn.

[CR14] Clarke A, Friese C, Washburn R (2015). Situational analysis in practice: Mapping research with grounded theory.

[CR15] Cleal B., H. Langstrup,. and J. Garfinkel. 2021. Living on the loop– agency, skill and (re)enchantment in DIY Artificial Pancreas System use. http://francisconunes.me/RealizingAIinHealthcareWS/papers/Cleal2021.pdf.

[CR106] Crocket Hamish (2019). Peer Mentoring in the Do-it-Yourself Artificial Pancreas System Community. Journal of Diabetes Science and Technology.

[CR16] Danesi G, Pralong M, Panese F, Burnand B, Grossen M (2020). Techno-social reconfigurations in diabetes (self-) care. Social Studies of Science.

[CR17] de Certeau M (1988). The practice of everyday life.

[CR18] DeLanda M (2001). Open-source: A movement in search of a philosophy.

[CR19] Demonaco HO, Torrance A, von Hippel C, von Hippel E (2019). When Patients Become Innovators. Mitsloan Management Review.

[CR20] Demonaco H, von Hippel E (2019). Patient-innovators fill gaps that industry hasn’t addressed — or can’t. Stat News.

[CR21] Dickinson JK, Guzman SJ, Maryniuk MD, O’Brian CA, Kadohiro JK, Jackson RA, D’Hondt N, Montgomery B, Close KL, Funnell MM (2017). The use of language in diabetes care and education. The Diabetes Educator.

[CR22] Duclos V, Criado TS (2020). Care in Trouble: Ecologies of Support from Below and Beyond: Medical Antropology Quarterly. International Journal for the Analysis of Health.

[CR23] Dumit J (2006). Illnesses you have to fight to get: Facts as forces in uncertain, emergent illnesses. Social Science & Medicine.

[CR24] Epstein S (1995). The construction of lay expertise: AIDS Activism and the forging of credibility in the reform of clinical trials. Science, Technology, & Human Values.

[CR25] Epstein S (1998). Impure science: AIDS, activism, and the politics of knowledge.

[CR26] Escobar A (2001). Culture sits in place: Reflections on globalism and subaltern strategies of localization. Political Geography.

[CR27] Falke O (2018). Der Patient als epistemische Größe: Praktisches Wissen und Selbsttechniken in der Diabetestherapie 1922–1960. Medizinhistorisches Journal.

[CR28] Farrington C (2017). Hacking diabetes: DIY artificial pancreas systems. The Lancet Diabetes and Endocrinology.

[CR29] Fiske A, Prainsack B, Buyx A (2019). Meeting the needs of underserved populations: Setting the agenda for more inclusive citizen science of medicine. Journal of Medical Ethics.

[CR30] Forlano, L. 2016. Hacking the Feminist Disabled Body. *Journal of Peer Production.* Special Issue on “Feminist (Un)Hacking.”

[CR31] Forno F, Graziano P (2019). From global to glocal: Sustainable Community Movement Organisations (SCMOs) in times of crisis. European Societies.

[CR32] Frickel S, Gibbon S, Howard J, Kempner J, Ottinger G, Hess DJ (2010). Undone science: charting social movement and civil society challenges to research agenda setting. Science, Technology & Human Values.

[CR33] Gallegos JE, Boyer C, Pauwels E, Kaplan WA, Peccoud J (2018). The open insulin project: a case study for 'Biohacked' medicines. Trends Biotechnology.

[CR34] Garfinkel, J. 2021. Diabetes as Illness and Metaphor: Stories from the Body-Technology" SCBE Seminar. https://mediaspace.stanford.edu/media/t/1_etfj95yq. Accessed 29 November 2021.

[CR35] Geiger S (2021). Healthcare activism. Markets, morals, and the collective good.

[CR36] Gottlieb SD, Cluck J (2019). “Going Rogue” re-coding resistance with type 1 diabetes. Culture & Society.

[CR37] Gottlieb SD, Geiger S (2021). The fantastical empowered patient. Healthcare activism: Markets, morals, and the collective good.

[CR38] Haraway D, Simians C (1991). A cyborg manifesto: Science, technology, and socialist-feminism in the late twentieth century. Cyborgs and women: The reinvention of nature.

[CR39] Hatch AR, Gordon JT, Sternlieb SR, Boero N, Manson K (2020). The artificial pancreas in cyborg bodies. The oxford handbook of the sociology of body and embodiment.

[CR40] Heinemann, L. 2017. Rolle der Diabetes-Technologie in der Diabetestherapie. In: Deutsche Diabetes Gesellschaft (ed.) Deutscher Gesundheitsbericht. Diabetes 2017: Bestandsaufnahme. Mainz: Kirchheim Verlag.

[CR41] Hendl T, Jansky B, Wild V, Loh J, Cockelberg M (2020). From design to data handling. why mHealth needs a feminist perspective. Feminist philosophy of technology.

[CR42] Hess DJ (2016). Undone science: Social movements, mobilized publics, and industrial transitions.

[CR43] Jansky B, Inthorn J, Seissing R (2021). Warum stechen, wenn man scannen kann?“: Zum Einsatz sensorbasierter Glukosemesssysteme in der Typ 1 Diabetestherapie. Digitale Patientenversorgung: Zur Computerisierung von Diagnostik, Therapie und Pflege.

[CR44] Jasanoff S, Kim SH (2013). Sociotechnical imaginaries and national energy policies. Science as Culture.

[CR45] Kaziunas, E. 2018. Designing for Lived Health: Engaging the Sociotechnical Complexity of Care Work. Ph.D. Dissertation: University of Michigan.

[CR46] Kaziunas E, Klinkman MS, Ackerman MS (2019). Precarious interventions: Designing for ecologies of care. Proceedings of the ACM on Human-Computer Interaction.

[CR47] Kaziunas, E., M.S. Ackerman, S. Lindtner, and J.M Lee. 2017 Caring through Data: Attending to the Social and Emotional Experiences of Health Datafication. Proceedings of the 2017 ACM Conference on Computer Supported Cooperative Work and Social Computing. Portland, Oregon, USA: Association for Computing Machinery.

[CR48] Kelty C (2005). Geeks, social imaginaries, and recursive publics. Cultural Anthropology.

[CR49] Kelty C (2008). Two Bits. The cultural significance of free Software.

[CR50] Kelty C (2013). There is no free software. Journal of Peer Production..

[CR51] Kesavadev J, Saboo B, Krishna MB, Krishnan G (2020). Evolution of insulin delivery devices: from syringes, pens, and pumps to DIY artificial pancreas. Diabetes Therapy.

[CR52] Kesavadev J, Srinivasna S, Saboo B, Krishna MB, Krishnan G (2020). The do-it-yourself artificial pancreas: A Comprehensive Review. Diabetes Therapy.

[CR53] King S (2004). Pink Ribbons Inc: Breast cancer activism and the politics of philanthropy. International Journal of Qualitative Studies in Education.

[CR54] Kingod N (2018). The tinkering m-patient: Co-constructing knowledge on how to live with type 1 diabetes through Facebook searching and sharing and offline tinkering with self-care. Health.

[CR55] Kish L, Topol E (2015). Unpatients-why patients should own their medical data. Nature Biotechnologies.

[CR56] Klawiter M (1999). Racing for the cure, walking women, and toxic touring: Mapping cultures of action within the bay area terrain of breast cancer. Social Problems.

[CR57] Latour B (2004). Why has critique run out of steam? From matters of fact to matters of concern. Critical Inquiry.

[CR58] Latour B (2005). Reassembling the social. An introduction to actor-network-theory.

[CR59] Lee JM, Hirschfeld E, Wedding J (2016). A patient-designed do-it-yourself mobile technology system for diabetes: promise and challenges for a new era in medicine. JAMA.

[CR60] Lehtiniemi T, Ruckenstein M (2019). The social imaginaries of data activism. Big Data & Society.

[CR61] Lewis D (2018). History and perspective on DIY closed looping. Journal of Diabetes Science and Technology.

[CR62] Lewis D, Leibrand S (2016). Real-world use of open source artificial pancreas systems. Journal of Diabetes Science and Technology.

[CR63] Lewis, D. 2019. Automated Insulin delivery. How artificial pancreas ‘closed loop’ systems can aid you in living with diabetes.

[CR64] Liggins AS (2020). Making diabetes. The politics of diabetes diagnostics in Uganda.

[CR65] Marcus GE (1995). Ethnography in/of the World System: The Emergence of Multi-Sited Ethnography. Annual Review of Anthropology.

[CR66] Marres N (2012). Material Participation: Technology, the environment and everyday publics.

[CR67] Mbali, M. 2005. TAC in the History of Rights-based, Patient driven HIV/AIDS Activism in South Africa. *Passages* 2. https://quod.lib.umich.edu/p/passages/4761530.0010.011/--tac-in-the-history-of-rights-based-patient-driven-hivaids?rgn=main;view=fulltext.

[CR68] Mol A (2009). Living with diabetes: Care beyond choice and control. The Lancet.

[CR69] Mol A, Law J (2004). Embodied action, enacted bodies: the example of hypoglycaemia. Body & Society.

[CR70] Moletsane R, Lesko N (2004). Overcoming paralysis: AIDS education-and-activism. Agenda.

[CR71] Murray, A.I. 2020. Biologics of resistance: The Open Insulin Project and the Promise of Antibiocapital. UC Santa Cruz.

[CR72] Nielsen KD, Langstrup H (2018). Tactics of material participation: How patients shape their engagement through e-health. Social Studies of Science.

[CR73] O'Donovan O (2007). Corporate colonization of health activism? Irish health advocacy organizations' modes of engagement with pharmaceutical corporations. International Journal of Health Service.

[CR74] O'Kane, A.A., A. Aliomar, R. Zheng, B. Schulte and G. Trombetta. 2019. Social, cultural and systematic frustrations motivating the formation of a diy hearing loss hacking community. Proceedings of the 2019 CHI Conference on Human Factors in Computing Systems. Association for Computing Machinery.

[CR75] Pearce J (2020). A review of open source ventilators for COVID-19 and future pandemics [version 2; peer review: 3 approved]. Research.

[CR76] Piras EM, Miele F (2017). Clinical self-tracking and monitoring technologies: Negotiations in the ICT-mediated patient–provider relationship. Health Sociology Review.

[CR77] Plotnick L, Henderson R (1998). Clinical management of the child and teenager with diabetes.

[CR78] Polk E, Servaes J (2014). Digital technology and construction of “Glocal” information flows. Social movements and social media in the age of sustainability. Technological determinism and social change: Communication in a tech-mad world.

[CR79] Prainsack B (2011). Voting with their Mice: personal genome testing and the “Participatory Turn” in disease research. Accountability in Research.

[CR80] Prainsack B (2014). The powers of participatory medicine. PLOS Biology.

[CR81] Prainsack B (2017). Personalized medicine: Empowered patients in the 21st century?.

[CR82] Rabeharisoa V (2006). From representation to mediation: The shaping of collective mobilization on muscular dystrophy in France. Social Science & Medicine.

[CR83] Rabeharisoa V, Moreira T, Akrich M (2014). Evidence-based activism: Patients’, users’A and activists’ groups in knowledge society. BioSocieties.

[CR84] Rabeharisoa V, Doganova L, Geiger S (2021). War on Diseases: patient organizations’ problematization and exploration of market issues. Healthcare activism: Markets, morals, and the collective good.

[CR85] Reiter H, Witzel A (2012). The problem-centred interview.

[CR86] Richterich A (2020). When open source design is vital: Critical making of DIY healthcare equipment during the COVID-19 pandemic. Health Sociology Review.

[CR87] Sánchez Criado T, Rodríguez-Giralt I, Mencaroni A (2015). Care in the (critical) making: Open prototyping, or the radicalisation of independent-living politics. Alter.

[CR88] Saunders B, Kitzinger J, Kitzinger C (2014). Anonymising interview data: Challenges and compromise in practice. Qualitative Research.

[CR89] Schicktanz S, Wehling P, Viehöver W, Keonen S (2015). The ethical legitimacy of patient organizations’ involvement in politics and knowledge production: Epistemic justice as conceptual basis. The public shaping of medical research.

[CR90] Schipp J, Skinner T, Holloway E, Scibilia R, Langstrup H, Speight J, Hendrieckx C (2021). How adults with type 1 diabetes are navigating the challenges of open-source artificial pancreas systems: A qualitative study. Diabetes Technology Therapy..

[CR91] Schultz, M. 2019. DIY Sleep Apnea Screening. url: https://medium.com/swlh/diy-sleep-apnea-screening-7e03c607c7e. Accessed 29 November 2021.

[CR92] Sharon T (2015). Healthy citizenship beyond autonomy and discipline: Tactical engagements with genetic testing. BioSocieties.

[CR93] Swyngedouw E, Cox K (1997). Neither local nor global: ‘Glocalization’ and the politics of scale. Spaces of globalization: Reasserting the power of the Local.

[CR94] Urkidi L, Walter M (2011). Dimensions of environmental justice in anti-gold mining movements in Latin America. Geoforum.

[CR95] Von Hippel E (2006). Democratizing innovation.

[CR96] von Hippel E (2009). Democratizing innovation: The evolving phenomenon of user innovation. International Journal of Innovation Science.

[CR97] Von Hippel E (2016). Free innovation.

[CR98] Wahlberg A (2018). Good Quality – the routinization of sperm banking in China.

[CR99] Wahlberg, A.L., M.A Jieun, A. Dokumaci, N. Kingod, M. Svensson, and L.L. Heinsen. 2021. Chronic living: Ethnographic explorations of daily lives swayed by (multiple) medical conditions. Somatosphere. http://somatosphere.net/2021/chronic-living.html/. Accessed 8 June 2021.

[CR100] Wehling P, Viehöver W, Koenen S (2015). The public shaping of medical research: Patient associations, health movements and biomedicine.

[CR101] Wiedeman L, Duttweiler S, Gugutzer R, Passoth J, Strübing J (2016). “Vom Piksen zum Scannen, vom Wert zu Daten“—Digitalisierte Selbstvermessung im Kontext von Diabetes. Leben nach Zahlen: Self-Tracking als Optimierungsprojekt?.

[CR102] Wilson CL, Flicker S, Restoule J-P, Furman E (2017). Narratives of resistance: (Re) telling the story of the HIV/AIDS movement—Because the lives and legacies of Black, Indigenous, and People of Colour communities depend on it. Health Tomorrow: Interdisciplinarity and Internationality.

[CR103] Williams S (2015). Digital defense: black feminists resist violence with hashtag activism. Feminist Media Studies.

[CR104] Zappavigna M (2015). Searchable talk: The linguistic functions of hashtags. Social Semiotics.

[CR105] Zhang, S. 2019. People are clamoring to buy old insulin pumps. How an obsolete medical device with a security flaw became a must-have for some patients with type 1 diabetes. The Atlantic.

